# Care pathways during a child’s final illness in rural South Africa: Findings from a social autopsy study

**DOI:** 10.1371/journal.pone.0224284

**Published:** 2019-10-22

**Authors:** Jessica Price, Merlin Willcox, Chodziwadziwa Whiteson Kabudula, Kobus Herbst, Lisa Hinton, Kathleen Kahn, Anthony Harnden

**Affiliations:** 1 Nuffield Department of Primary Care Health Sciences, University of Oxford, Oxford, England, United Kingdom; 2 Department of Primary Care and Population Sciences, University of Southampton, Southampton, England, United Kingdom; 3 MRC-Wits Rural Public Health and Health Transitions Research Unit (Agincourt), School of Public Health, Faculty of Health Sciences, University of the Witwatersrand, Johannesburg, South Africa; 4 Africa Health Research Institute, KwaZulu-Natal, South Africa; 5 School of Public Health, Faculty of Health Sciences, University of the Witwatersrand, Johannesburg, South Africa; Medical Research Council, SOUTH AFRICA

## Abstract

**Background:**

Half of under-5 deaths in South Africa occur at home, however the reasons remain poorly described and data on the care pathways during fatal childhood illness is limited. This study aimed to better describe care-seeking behavior in fatal childhood illness and to assess barriers to healthcare and modifiable factors that contribute to under-5 deaths in rural South Africa.

**Methods:**

We conducted a social autopsy study on all under-5 deaths in two rural South African health and demographic surveillance system sites. Descriptive analyses based on the Pathways to Survival Framework were used to characterise how caregivers move through the stages of seeking and providing care for children during their final illness and to identify modifiable factors that contributed to death.

**Findings:**

Of 53 deaths, 40% occurred outside health facilities. Rates of antenatal and perinatal preventative care-seeking were high: over 70% of mothers had tested for HIV, 93% received professional assistance during delivery and 79% of children were reportedly immunised appropriately for age. Of the 48 deaths tracked through the stages of the Pathways to Survival Framework, 10% died suddenly without any care, 23% received home care of whom 80% had signs of severe or possibly severe illness, and 85% sought or attempted to seek formal care outside the home. Although half of all children left the first facility alive, only 27% were referred for further care.

**Conclusions:**

Modifiable factors for preventing deaths during a child’s final illness occur both inside and outside the home. The most important modifiable factors occurring inside the home relate to caregivers’ recognition of illness and appreciation of urgency in response to the severity of the child’s symptoms and signs. Outside the home, modifiable factors relate to inadequate referral and follow-up by health professionals. Further research should focus on identifying and overcoming barriers to referral.

## Introduction

Over 6 million children die annually before their 5^th^ birthday, with a growing proportion occurring in sub-Saharan Africa [1]. Despite South Africa’s relative wealth in the region and progressive health policies including free public healthcare for children under six years, an expanded vaccination schedule, Prevention of Mother-to-Child Transmission (PMTCT) programmes and promotion of the Integrated Management of Childhood Illness (IMCI) guidelines, mortality in children younger than five years remains high at 37/1000 live births in 2017 [2]. Achieving the Sustainable Development Goal 3.2 of reducing the mortality rate in children younger than five years to 25/1000 live births by 2030 requires innovative policies that focus not only on biological interventions but also on increasing access to high quality healthcare services. While classification of biological causes of death is improving due to improved vital registration systems, facility-based audits and verbal autopsies used in health and demographic surveillance systems, there remains limited information on healthcare usage patterns, barriers to access, critical causes of delay in care-seeking, and quality of healthcare in South Africa, particularly in rural areas. This is especially the case in children who die at home, which constitute over half of deaths in children younger than five years [3,4].

Social autopsies are increasingly recognised as a means of identifying important information on modifiable factors in child deaths. Social autopsies are structured, standardised interviews with the caregiver of the deceased which attempt to make a “social diagnosis” of cause of death by identifying its cultural, social and health-systems antecedents [5–7]. As such, social autopsies “provide evidence in the form of actionable data to communities, health programmes, and health policymakers, and increase motivation at all levels to take appropriate and effective actions” [6]. A series of standardized social autopsy tools have been developed, including the International Network for the Demographic Evaluation of Populations and Their Health(INDEPTH) Social Autopsy tools for neonatal and child deaths (accessible at http://www.indepth-network.org/resources/tools) [5]. These questionnaires are structured to enable analysis based on the BASICS/CDC Pathways to Survival Framework [5,8], and have been used in multiple settings across sub-Saharan Africa [5].

Social autopsies for under-5 deaths conducted across sub-Saharan Africa have highlighted deficits in illness recognition, availability of transport to and between health facilities, and insufficient referral pathways [5,9–12]. However, it is unclear whether the same is true in South Africa. Currently data on modifiable factors contributing to perinatal and child deaths in South Africa are drawn from facility-based audits, namely the Perinatal Problem Identification Programme, and the Child Health Problem Identification Programme [13,14]. While such audits are critical to assessing quality of healthcare at facilities, and identifying delays which may contribute to death, they only capture data on children who died in health facilities, and only offer the perspective of healthcare workers. Recent research that included home deaths suggested that the majority of children who died at home were taken to a formal healthcare provider during their final illness, suggesting that home deaths are not merely a function of caregivers’ failure to recognise signs of illness and further highlighting the need to better understand modifiable factors that contribute to home deaths [3]. Social autopsies, as a population-based data collection tool, provide information on all deaths, including those that occur outside health facilities—at home or in the community, and identify barriers to healthcare from caregivers’ perspectives.

This study aimed to better describe care-seeking behavior in fatal childhood illness and to assess barriers to healthcare and modifiable factors that contribute to deaths in children younger than five years in rural South Africa using a social autopsy nested within established verbal autopsy data collection systems.

## Methods

This was a population-based, cross-sectional verbal and social autopsy (VASA) study, conducted in two health and demographic surveillance system (HDSS) sites in rural South Africa, the Agincourt HDSS and the Africa Health Research Institute (AHRI). Both sites are members of the INDEPTH Network (www.indepth-network.org) and are two of the pioneer nodes of the South African Population Research Infrastructure Network (SAPRIN) (http://saprin.mrc.ac.za).

### Study sites

The Agincourt and AHRI HDSS sites are situated in poor rural areas of South Africa, with limited infrastructure. Together these sites cover more than 280 000 people in over 40 500 households [15]. Both sites have high levels of temporary labour migration (33–36%) with household members oscillating between their place of work and their rural home [16–18]. The majority of households are dependent on income from social grants (particularly government pensions for older adults and child grants). Mozambican immigrants account for a third of the population under surveillance in Agincourt.

In Agincourt, healthcare is provided by seven primary care nurse-led clinics, 10 private general practitioners (GPs) and two health centres. Only the two health centres remain open 24 hours and include emergency services. Three district hospitals are accessible 25-60km away [17]. In AHRI, healthcare access is via 10 clinics, six private GPs all located in Mtubatuba, and one health centre. Only the health centre is open 24 hours. The nearest district hospital is 45 km away, on the other side of a nature reserve where wild animals roam free. There are also approximately 300 traditional healers operating in and around each study site [19].

### Sampling strategy

We included all under-5 deaths identified during the 2017 household surveys across the two HDSS sites in this study.

### Data collection

#### Household surveys

The Agincourt HDSS has conducted annual household surveys of key demographic and health data since 1992, while AHRI’s population health surveillance started in 2000 and now involves four-monthly monitoring. The household surveys include identifying any new pregnancies, births and in-migrations, as well as all deaths and out-migrations.

Data on household and maternal characteristics were taken from the household surveys. Socioeconomic status was determined based on household asset ownership: households were divided into quintiles from 1 (poorest) to 5 (least poor). In Agincourt an absolute asset index is used to rank households as described by Kabudula et al [20,21], before assigning the household to a socioeconomic quintile. In AHRI, a wealth index is derived using principal component analysis as described by Nyirenda et al [22]. Households are assigned a wealth score which follow a standard normal distribution, and subsequently divided into quintiles. The socioeconomic quintile of each household was determined relative to all other households in their HDSS in the year that the child died.

#### Verbal and social autopsy

All deaths of members of the HDSS sites that are identified during the household survey are followed up and investigated using verbal autopsies (VA)–a structured interview with the caregiver of the deceased to determine biological cause of death. Verbal autopsies have been validated as a means of establishing cause of death in a rural South African population [23]. Both sites use the standardized World Health Organization VA tool, which has included 10 questions on circumstances of death since 2012 [24].

We added a locally-relevant adaptation of the INDEPTH Network Social Autopsy tools for neonatal and child deaths [5] to the WHO 2016 VA tools for neonatal and child deaths. The social autopsy questions focus on the specific actions taken by caregivers during the child’s final illness and broadly follow the stages of the care pathway: 1) identifying symptoms, 2) providing care inside the home, 3) seeking healthcare outside the home 4) determining they were referred for further care and 5) whether they accepted that referral. In addition, the social autopsy attempts to identify barriers faced in accessing healthcare at each stage of the care-seeking process (see S1 Table for the adapted social autopsy tool that was integrated into the WHO 2016 VA).

Interviewers were trained in the use of the verbal and social autopsy tools, which were translated into isiZulu and Shangaan, the local languages in the two HDSS sites, and back-translated into English to ensure accuracy. The verbal and social autopsy interviews were conducted between July 2017 and February 2018 for all deaths in children younger than five years identified across the HDSS sites in the 2017 household surveys. Interviews were conducted at least one month after the death to allow for the customary mourning period to pass, and up to 18 months after the death where special arrangements were required to ensure the VA interviewers were able to interview the primary caregiver of the deceased who would be the most appropriate respondent.

### Data analysis

Descriptive data analysis was conducted using the BASICS/CDC Pathways to Survival Framework [25]. Data was pooled across the two sites to give a more representative picture of rural South Africa. However, where any differences were found, we have highlighted results for each site. Neonates that were born and died in health facilities without discharge were excluded from analysis. The sociodemographic characteristics of participants and households were reported to provide relevant context for the barriers to access and modifiable factors identified during the social autopsy interviews, however given the small number of deaths in 2017, we did not perform bivariate or multivariate analyses of associations between sociodemographic characteristics and care-seeking patterns.

### Ethics

This study was approved by the University of Oxford Medical Sciences Inter-divisional Research Ethics Committee (R52414/RE001), the University of Witwatersrand Human Research Ethics Committee (M1705102) and the University of KwaZulu Natal Biomedical Research Ethics Committee (BE 290/16). Written consent was obtained from the household head for participation in the household survey. The verbal autopsy interviewers took written consent separately from primary caregiver of the deceased to participate in the verbal and social autopsy interview.

## Results

### Sociodemographic characteristics of deceased children and their households

Overall, 53 children younger than five years died in 2017 in both Agincourt and AHRI, of which 12 (23%) were neonatal deaths (9 early neonatal deaths between 0–7 days of life, 3 late neonatal deaths between 8–27 days of life), and a further 17 (32%) were infant deaths between 1–11 months. Forty percent of deaths (21 children) occurred outside health facilities, and of those deaths outside health facilities 70% occurred at home (15 children). Four children (8%) died on route to a provider, of whom 3 died on route to the first provider. A higher proportion of deaths occurred at home in AHRI (38%), compared to Agincourt (21%), though this was not statistically significant (p = 0.176). Over half (31 (58%)) of verbal and social autopsy respondents were parents, 16 (30%) were family members in the same household as the child, 1 (2%) was another relative not in the same household, 1 (2%) was a friend of the family, 3 (6%) had other relationships to the deceased and in 1 case (2%) the respondent’s relationship to the child was unspecified. Table 1 provides a full description of the sociodemographic characteristics of the deceased children, overall and by study site.

**Table 1:** Sociodemographic characteristics of deceased children in both study sites, 2017.

	Total n = 53		AHRI n = 24		Agincourt n = 29		P value
	n	%	n	%	n	%	
Age group							0.469
0–27 days	12	23	7	29	5	17	
1–11 months	17	32	6	25	11	38	
12–59 months	24	45	11	46	13	45	
Median age at death, in months (IQR):	10 (1–26)	-	8 (0–25)	-	11 (2–29)	-	0.362
Sex							
Female	27	51	12	50	15	52	
Male	26	49	12	50	14	48	
Masculinity ratio (Boy/Girl x100)	96	-	100	-	93	-	0.350
Place of birth							0.885
Hospital	43	81	19	79	24	83	
Other health facility	3	6	1	4	2	7	
Home	2	4	1	4	1	3	
On route to provider	0	0	0	0	0	0	
Other	0	0	0	0	0	0	
Unknown	5	9	3	13	2	7	
Place of death							0.490
Hospital	26	49	9	38	17	59	
Other health facility	4	8	2	8	2	7	
Home	15	28	9	38	6	21	
On route to provider	4	8	1	4	3	10	
Other	2	4	1	4	1	3	
Unknown	2	4	2	8	0	0	
Child’s immunisation status							
Child immunised (self-reported)	42	79	17	71	25	83	0.225
Child vaccination card seen by interviewer	9	17	4	17	5	17	0.154

Table 2 details the sociodemographic characteristics of the mothers and households of the deceased children. All 53 mothers were still alive at the time of their child’s death. Over 80% of mothers (43/52) were resident in the household, while 17% (9/52) were temporary migrants; in 1 case migrancy status of the mother was unreported. Migrancy rates in AHRI (25%) appeared to be double that of Agincourt (11%), though this was not statistically significant (p = 0.174). Over 20% of mothers (11/53) were reported to be HIV positive and 1 child was reported as HIV positive. The median household size was 10 people, with a median of 2 children younger than five years per household. Overall, deaths were fairly evenly distributed across socioeconomic quintiles, however this distribution differed between the sites. In Agincourt, 83% of deaths in this sample were from households in the lowest 3 socioeconomic quintiles—only 17% of deaths occurred in households that fell into the less poor and least poor quintiles. In contrast, in AHRI 66% of deaths occurred in households that were in the top two socioeconomic quintiles (less poor/least poor), and no deaths occurred in households in the poorest quintile.

**Table 2:** Sociodemographic characteristics of the households of deceased children in both study sites, 2017.

	Total (n = 53)		AHRI (n = 24)		Agincourt (n = 29)		P value
	n	%	n	%	n	%	
Maternal characteristics							
Mother Alive	53	100	24	100	29	100	-
Mother residency status at time of death							0.175
Resident household member	43	83	18	75	25	89	
Temporary migrant	9	17	6	25	3	11	
Mother’s highest level of education							0.071
None	5	9	3	13	2	7	
Started/completed primary school	3	6	0	0	3	10	
Started/completed high school	36	68	14	58	22	76	
Tertiary education	6	11	5	21	1	3	
Unknown/unreported	3	6	2	8	1	3	
Mother employed	8	15	5	21	3	10	0.264
Mother’s HIV status*							0.148
Positive	11	21	7	29	4	14	
Negative	34	64	12	50	22	76	
Unknown/unreported	8	15	5	21	3	10	
Mother’s nationality**	(Not totalled)						-
South African			-	-	17	59	
Mozambican descent			-	-	8	28	
Unknown			-	-	4	14	
Household characteristics							
Median Household size (IQR) (including resident and non-resident members)	10 (6–13)	-	11 (8–18)	-	9 (5–11)	-	0.010
1–5 members	8	15	1	4	7	24	
6–10 members	20	38	9	38	11	38	
11–15 members	14	26	6	25	8	28	
>15 members	11	21	8	33	3	10	
Median number of children under 5 years in the household (IQR)	2 (1–3)	-	2 (1–2)		2 (1–3)	-	0.089
Median number of household members employed (IQR)	1 (1–3)	-	2 (1–3)		1 (0–3)	-	0.057
Household head female	21	40	9	38	12	43	0.712
Household SES							<0.001
Poorest	9	17	0	0	9	32	
Poor	11	21	4	17	7	25	
Medium	12	23	4	17	8	29	
Less poor	14	27	13	54	1	4	
Least poor	6	12	3	13	3	11	
Mean distance to nearest clinic in km (SD)	2.56 (1.77)	-	2.70 (1.61)	-	2.44 (1.92)	-	0.599

*HIV status: the gold standard at AHRI was the recorded HIV status based on home testing. If this was unavailable, we used the HIV status as reported in the verbal autopsy. In Agincourt, all HIV data is as reported in the verbal autopsy.

**Immigration status only recorded in Agincourt. Assessment of immigration status was based first on the household classification in the Agincourt Household Data, and if unknown, then on reported nationality or former refugee status in answer to the verbal autopsy questionnaire.

## Preventive care-seeking

Antenatal and perinatal care-seeking was consistent across the two sites. Over 70% (38/53) of mothers had reportedly been tested for HIV at least once, 7/53 (13%) reported the mother was never tested, and in 5 cases (9%) the respondent was unsure. Forty-seven mothers (89%) received professional assistance during delivery, including one who delivered at home; three did not and five were missing data.

In total, 42/53 children (79%) were reportedly immunised appropriate for their age, though only 21 (40%) respondents reported having a vaccination card, and of those only 9 (17%) were available to be seen by the VA interviewer (Table 1).

### Pathways to survival (curative care-seeking)

**Fig 1:**
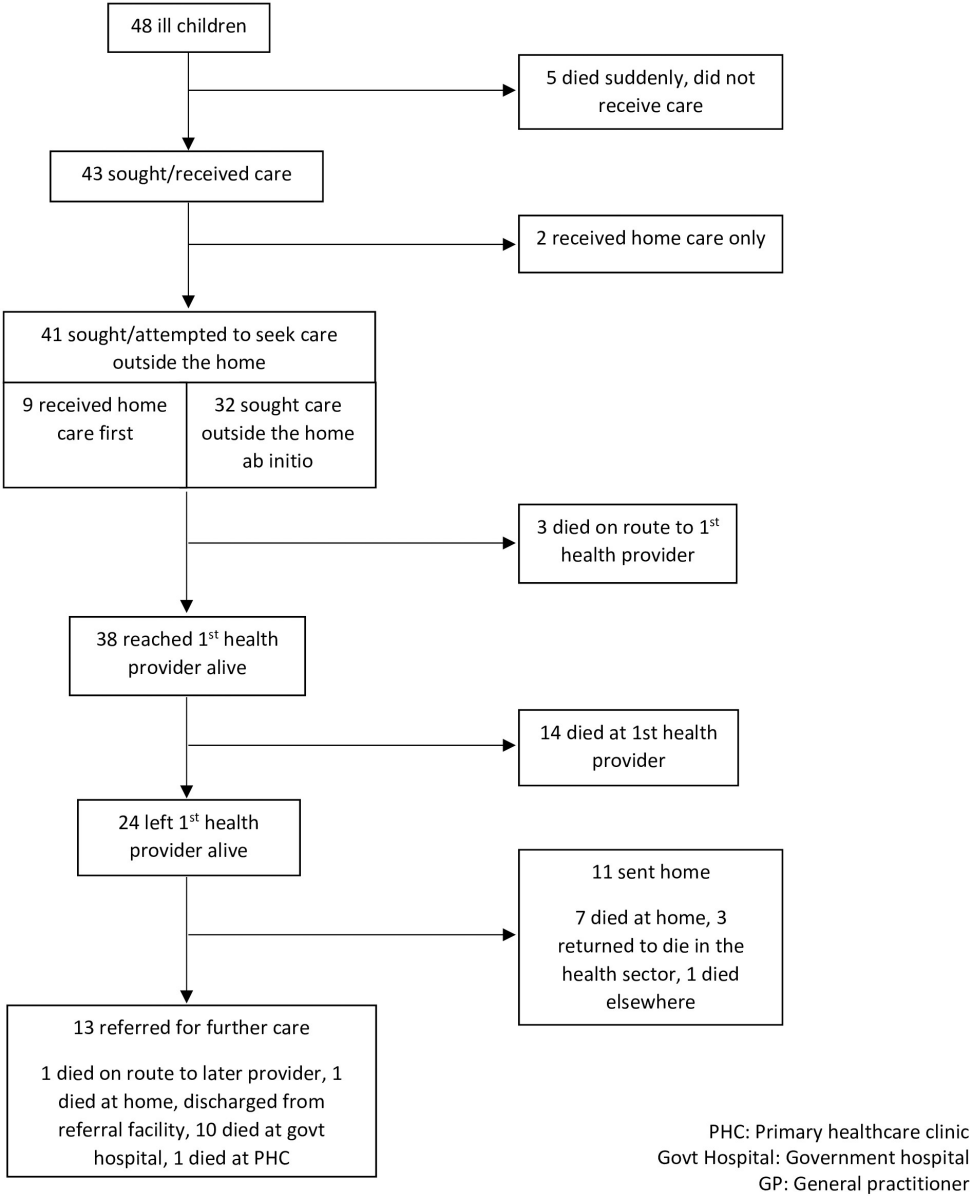
Care pathways of 48 children who died in rural South Africa.

Of the 53 deaths, five were excluded from further analysis, including one child who was born and died in a health facility without being discharged, and four deaths where no care-seeking data were available. Fig 1 shows the care pathway for the remaining 48 deaths.

#### Within the home

Of the 48 deaths, five children (10%) died suddenly without receiving any care. Caregivers of two of the five children did not identify any signs of illness. Of the remaining 43 deaths, 39 children had signs of illness including 34 with signs of severe or possibly severe illness; 4 caregivers did not recall any symptoms of illness. Home care was given to 11 children (23%), including nine children with at least one severe or possibly severe symptom. All those given home care reportedly received western medicine; one also received traditional medicine.

#### Seeking healthcare outside the home

Forty-one caregivers (85%) sought or attempted to seek healthcare for their child outside the home. The median delay to seeking healthcare from the first provider was 0 days (IQR 0–1 day), however four caregivers of children with a severe or possibly severe illness delayed more than 24 hours to seek healthcare outside the home. Thirty-eight children (93% of those taken outside the home for care) arrived at a formal healthcare provider alive; three children died on route to the first provider.

Twenty-four children left the first provider alive (50% of all deaths, and 63% of those arriving at the provider alive). Only 13 of these children were referred to another facility (27% of all deaths, 54% of those leaving the facility alive) (Fig 1). However, referral rates varied significantly between the study sites; in AHRI only two children were referred out of 10 who left the first provider alive (20%), whereas 11 of the 14 children leaving the first provider alive in Agincourt were referred (79%) (p = 0.004). Reasons for referral included 1) for better care (7 children), 2) due to a lack of equipment (3 children), and 3) the child was deteriorating (2 children). Almost all caregivers (12/13) accepted the referral for further healthcare. Of those children not referred, three sought care again of their own accord and died in a health facility, one died elsewhere and seven subsequently died at home (15% of all deaths, 47% of home deaths, 64% of those not referred). The median total duration of illness was two days (IQR 0–4 days).

All children taken outside the home for healthcare sought care from a formal public or private healthcare provider (GP/public clinic/hospital). Over half of children who attended a formal healthcare provider sought care from a primary care clinic first (Fig 1). No child was reportedly taken to a traditional healer. Minimal difference was found in the proportion of children who sought or attempted to seek healthcare based on age group (90% of neonates taken for healthcare and 82% of infants and young children), or HIV-status of the mother (80% of children of HIV-positive mothers were taken for healthcare compared to 83% of children of HIV-negative mothers).

#### Seeking healthcare from multiple providers

Whether by referral or self-presentation, 16 children (33%) went to a second provider: four went to primary care clinics, 11 to government hospitals and one to a private GP. Overall, 18 children were taken to a single provider (excluding the three who died on route to the first provider), 12 to two providers, three to three providers and one to four providers. This excludes the two children who died in health facilities without specifying additional information about the providers accessed.

#### Place of death and care-seeking behaviour

Overall 42% of children (20/48) died outside the health facility; 31% (15/48) at home, 8% (4/48) on route to a healthcare provider, and 2% (1/48) elsewhere. Twenty-one percent of children (10/48) died without receiving formal healthcare, including seven children who died at home and three children who died on route to the first health provider. Seventeen percent (8/48) of children died at home after receiving healthcare from a formal health provider. Table 3 presents a breakdown of the demographic and socioeconomic characteristics of those children tracked through the care pathway based on place of death.

**Table 3:** Sociodemographic characteristics of children who died at home, in a health facility, on route and elsewhere.

	Died at home		Died in a health facility		On route		Elsewhere		P value
	n = 15	%*	n = 28	%*	n = 4	%*	n = 1	%*	
Age group									0.698
Neonate	1	7	8	29	1	25	0	0	
1–11 months	6	40	8	29	2	50	1	100	
1–4 years	8	53	12	43	1	25	0	0	
Socioeconomic status									0.184
Poorest	1	7	4	14	3	75	1	100	
Poor	3	20	6	21	0	0	0	0	
Medium	3	20	9	32	0	0	0	0	
Less poor	6	40	5	18	0	0	0	0	
Least poor	2	13	3	11	1	25	0	0	
Mothers education									0.109
None	2	13	3	11	0	0	0	0	
Primary	0	0	2	7	0	0	1	100	
Secondary	11	73	18	64	3	75	0	0	
Tertiary	2	13	3	11	1	25	0	0	
Mother HIV status									0.644
Negative	10	67	17	62	3	75	1	100	
Positive	4	27	6	21	0	0	0	0	
Don’t know	1	7	5	18	1	25	0	0	
Mother residency									0.855
Resident	13	87	22	79	4	100	1	100	
Migrant	2	13	5	18	0	0	0	0	

*percentages may not sum to 100 due to rounding

## Discussion

This study aimed to characterise the processes for seeking and providing healthcare for children dying below the age of five years as a means of identifying modifiable factors that contribute to child deaths and which might serve as targets for intervention. The findings in this study reaffirmed that almost half of all deaths in children younger than five years occurred outside of health facilities [3,12], including 28% of deaths that occurred at home, and that the large majority of children were taken for healthcare during their final illness. Results highlighted three groups of children, with corresponding modifiable factors within and outside the home that contributed to death: first, children who died before entering the health sector; second, children who died shortly after entry to the health sector; and third, children who died at home after receiving formal healthcare.

### Modifiable factors within the home

Most caregivers recognised illness, and two thirds recognised signs of severe or possibly severe disease. This is in keeping with rates of symptom recognition found elsewhere in sub-Saharan Africa [12,26]. However, 21% of children died without receiving formal healthcare and a quarter of children with severe or possibly severe symptoms (9/34) were given home treatment before being taken for formal healthcare. The caregivers of four children with severe or possibly severe symptoms delayed more than one day before seeking healthcare. This suggests that some caregivers may not have fully appreciated the severity of the child’s symptoms and the urgency required in seeking healthcare. Late recognition of illness or failure to appreciate the urgent need for care contributes to deaths of children who do not enter the formal health sector, and to those who die soon after entering health facilities. Similar concerns have been noted in other studies, which have suggested that contributing factors include underlying low levels of maternal education, low health literacy and differences in cultural interpretations of symptoms and causes of illness [11,27–29]. In this study the distance to the nearest health facility was low (average 2.5km), and so proximity is unlikely to have been a significant barrier to accessing healthcare during the day. However, after hours, caregivers would have needed to travel to one of the health centres which are open 24 hours and are further away. Therefore, distance and lack of transport may have prevented caregivers from accessing healthcare services at night. This may have contributed to deaths in all three groups of children, regardless of their place of death, possibly preventing caregivers from accessing care for the first time, delaying their access until the following day when the child might have been more severely ill, or preventing them from re-seeking care at a later stage.

### Modifiable factors outside the home

Both preventive and curative care-seeking patterns suggest broadly high rates of access to formal healthcare services. Over three quarters of caregivers reported that the child was vaccinated, with no difference noted based on place of death, and 79% of children were seen by a formal healthcare provider during the final illness, including half of children who died at home. All children reportedly used only formal healthcare providers, and about a third of children were taken to more than one provider. However, referral rates from primary care facilities to higher levels of care were low (54% overall and only 20% in AHRI).

Referral might be an appropriate metric for quality of healthcare at primary care clinics, as it may reflect the ability of healthcare workers to recognise signs of severe illness and also the degree of safety-netting or follow-up care that is in place. This is especially important given the short duration of the final illness episode (median illness duration 2 days, IQR 0–4 days), suggesting that while it is possible that children were seen when they were still relatively well, it is unlikely to account for the failure to refer in all cases. Low referral rates have been reported in other social autopsy studies, particularly for neonates, where poor training in healthcare for the sick newborn has been suggested as an important contributing factor [28]. However, little research has focused specifically on identifying barriers to referral. Poor quality of care, low referral rates and a lack of safety-netting or follow-up are modifiable factors that contribute to home deaths in children who have received formal healthcare.

There was a significant difference in referral rates between the two study sites which might best be explained by differences in the barriers to referral. On the one hand, if referral is a marker of healthcare quality, this difference might reflect differences in the quality of healthcare in the two provinces, which were noted in the 2014/15 South African District Health Barometer report [30]. However, another possible explanation for this difference is the geography of the AHRI study site. Clinics serving this community are separated from the main referral hospital by a wildlife conservation area. This area is poorly lit and generally considered unsafe to cross at night, even in a car, as wild animals roam free. Previous research has highlighted delays in ambulance services in reaching clinics to transfer patients to the hospital [31–33], which may add to the difficulty of referring children. Further research is needed to explore this hypothesis.

Differences in the distribution of death across socioeconomic quintiles in the two study sites might also point to modifiable factors outside the home. In AHRI, 66% of all deaths occurred in the top two socioeconomic quintiles, compared to only 17% in Agincourt. This is a small sample and would not necessarily be representative of long-term trends in the population. However, it is possible that the pattern of under-5 deaths across socioeconomic quintiles in AHRI is representative of patients who initially access private providers (general practitioners or paediatricians), paying for services with out-of-pocket payments. However, caregivers run out of money if the child needs to be hospitalised or required follow-up as they don’t have medical aid or hospital plan cover. This group may also be increasingly sceptical of the quality of care in government facilities and so delay going to the public sector services even when they can no longer afford private care. These children fall into the gap between private and public health services. Further research is required to fully explain this phenomenon, which might be of particular interest as South Africa explores a National Health Insurance policy.

#### Care-seeking by age group

We did not find a difference in care-seeking patterns for neonates and infants and young children. This is in contrast to a recent review of social autopsies from across sub-Saharan Africa which showed much lower care-seeking rates for neonates—both as an absolute rate (approximately half of neonates taken for healthcare in sub-Saharan Africa compared to 70% in this study) and when compared to infants and young children (care-seeking for neonates was lower than for children 1–59 months in the same region) [12]. The higher rates of care-seeking overall may reflect greater ease of access to healthcare facilities in South Africa—even in rural areas—compared to other sub-Saharan African countries.

The high in-facility delivery rates might also explain the higher rates of neonatal care-seeking in South Africa compared to elsewhere in sub-Saharan Africa. All neonates tracked through the care pathway had been discharged from the health facility where they were born. It is likely that children who are premature, low birthweight or ill after birth are identified in the facilities during routine post-natal checks and admitted for further care. This means that those children discharged after delivery are likely born at term and mostly healthy newborns with better survival chances, and in whom it is slightly easier for caregivers to identify signs of illness. In this study, recognition of signs of illness amongst neonates was high with only one 27 days old neonate having died without the caregivers identifying any signs of illness. Term, mostly well neonates, also have more reserves to survive slightly longer after developing signs of illness, giving caregivers extra time to seek healthcare. In contrast, in settings with high home delivery rates, there might be a greater delay in recognising signs of distress soon after birth, deciding to seek healthcare and in being able to reach a healthcare facility resulting in lower care-seeking rates and more home deaths.

#### Choice of healthcare provider

Public healthcare providers were used much more frequently than private providers: 90% of caregivers sought healthcare for their child from a government provider (either primary care clinic or hospital) as their first provider, and all referrals were to a government healthcare facility. Only six caregivers (13%) reported utilizing the private sector, of which four (67%) went to a GP. While the high rates of care-seeking from government facilities may suggest relatively easy access to healthcare services, this finding might also reflect financial barriers which limit access to private GPs (whereas government healthcare is free). This is of concern as government clinics are notorious for having long waiting times, medication stockouts, variable quality of care and poor staff attitudes [31,34,35]. Therefore, while rates of use are high, further research is required to determine whether this figure is a marker of good access to healthcare, or a function of lack of access to better quality health services.

### Limitations

There are a number of important limitations to this study. The first relates to the possibility of missing neonatal deaths. This is particularly the case in Agincourt where data collection happens annually, and might partially account for a lower percentage of neonatal deaths (17% of the under-5 deaths in this sample compared to 29% in AHRI). Whilst at each household census, the household head is asked about all pregnancies, births and deaths, it is possible that a full episode (pregnancy, birth and neonatal death) could be missed if it fell between census update rounds and was not reported by the household respondent. However, there are several safeguards in place to reduce the risk of missing neonatal deaths, including capturing pregnancies independently and training census fieldworkers to probe specifically for information about all births and neonates. Second, the social autopsy tool is, by definition, a methodology that captures the perspectives of the caregiver of the deceased. While this is an advantage in understanding caregiver perceptions around barriers to accessing healthcare, we were unable to assess the quality of healthcare received, and the appropriateness of healthcare worker decisions related to medication choices, referral or follow-up guidance. Findings on quality of healthcare from health facility audits in the area should therefore supplement the social autopsy results. Additional qualitative work would also be valuable in further exploring the barriers identified by caregivers. Also, the social autopsy relies on caregivers to honestly self-report on behavior. However, it is likely that the rate of traditional medicine use, and consultation of informal healthcare providers was underreported as HDSS teams are still seen as conducting “western research” and communities may fail to report practices which would be undesirable or criticised in those circles—such as the use of traditional medicine [36]. This is even more likely when nurses, clearly practitioners of “western medicine” conduct the interviews as is the case in AHRI. By this same logic, it is possible that care-seeking from formal providers is overreported by caregivers. Third, we relied on self-reporting to collect vaccination status as very few vaccination cards were available to be reviewed. This is partly due to cultural practices of burying the vaccination card with the deceased child (as part of their possessions). It is likely that self-reporting overestimates the vaccination rates, and fails to account for children who were only partially vaccinated (immunisations were not up to date given their age). For accurate vaccination data one would need to audit live children whose vaccination cards are still accessible. Fourth, this research included only caregivers of children who had died. Conducting similar interviews with caregivers of children who survived a recent illness would provide a better description of care pathways in child illness more generally and allow us to contrast care pathways between those children who survived and those who died. Finally, while the decline in under-5 mortality means that there are fewer deaths in each study site, the resultant small study size meant that it was not possible to conduct bivariate or multivariate analysis to assess associations between household, maternal or child characteristics and care-seeking behaviors during a child’s final illness. As subsequent years of social autopsy data are collected, such analysis would be of value.

### Policy implications

Home deaths are a particular concern in South Africa, and efforts to reduce under-5 mortality need to effectively target this vulnerable group. Based on findings from this study, policies should focus on addressing factors within and outside the home. Within the home, improved caregiver recognition of danger signs and appreciation of the urgency of care-seeking in the presence of signs of severe or possibly severe illness is required. Known effective community-based delivery of educational interventions include using facilitator-led participatory women’s groups [37,38], routine home visits to pregnant and newly-delivered mothers [39,40], community-based workers teaching mothers about signs and symptoms of illness and diversifying the messaging techniques used to deliver specific health messages to the whole community [41]. Similar interventions including care groups [42] and home visits by community health workers have been trialled in South Africa [43], and have been shown to be feasible and effective.

However, improved illness recognition alone is insufficient, as demonstrated by the finding that over 50% of children who died at home were taken outside the home for healthcare during their final illness. Outside the home, quality of healthcare concerns should be fully evaluated using the existing health facility audits, and appropriate interventions supported to target the modifiable factors identified. Particular attention should also be paid to identifying and understanding barriers to referral, which fall outside the ambit of facility audits. Guidelines for safety-netting or follow-up appointments should be clarified, adherence to guidelines assessed, and structural barriers identified.

Finally, further research is required to understand not just where, but why children are lost from formal care pathways looking at both supply- and demand-side factors. Qualitative research would be especially valuable to better understand why caregivers chose one provider over another, the impact of caregivers’ experiences of healthcare on subsequent decision-making during their child’s illness, and how caregivers overcame barriers to healthcare.

However, improved illness recognition alone is insufficient, as demonstrated by the finding that over 50% of children who died at home were taken outside the home for healthcare during their final illness. Outside the home, quality of healthcare concerns should be fully evaluated using the existing health facility audits, and appropriate interventions supported to target the modifiable factors identified. Particular attention should also be paid to identifying and understanding barriers to referral, which fall outside the ambit of facility audits. Guidelines for safety-netting or follow-up appointments should be clarified, adherence to guidelines assessed, and structural barriers identified.

Finally, further research is required to understand not just where, but why children are lost from formal care pathways looking at both supply- and demand-side factors. Insights into the impact of caregivers’ experiences of healthcare on subsequent decision-making in relation to their child’s illness, and how caregivers overcame barriers to healthcare are critical to incorporate into the design of all interventions.

## Conclusions

Children who die at home represent a vulnerable group whose access to quality healthcare has been inadequate. Despite good overall access to healthcare, with high rates of care-seeking across all age groups in rural South Africa, modifiable factors exist within and outside the home. Within the home, improved caregiver recognition of signs of illness is still needed, particularly to reduce home deaths. However, this alone is insufficient—outside the home parallel efforts are required to adequately retain children in formal care pathways with particular attention paid to referral and follow-up healthcare services.

## Supporting information

S1 Table Adapted Social Autopsy Tool. (DOCX)
