# Supplementary material for: Care pathways during a child’s final illness in rural South Africa: Findings from a social autopsy study
Source: PLoS One. 2019 Oct 22;14(10):e0224284. doi: 10.1371/journal.pone.0224284 (PMC6804973; doi:10.1371/journal.pone.0224284)
Supplement: S1 Table — (DOCX) [file pone.0224284.s001.docx]

**S1 Table: Adapted Social Autopsy Tool**

| **Pathway to Survival stage** | **Question** | **Answers** | |
| --- | --- | --- | --- |
| Care inside the home | 1. a. Was care initiated in the home? 2. How much time after first symptom was recognised was care initiated in the home? | Yes…………………………………………………………………  No………………………………………………………………….  Don’t Know……………………………………………………  Days………………………………………………………………  Don’t know…………………………………………………… | □  □  □  □□  □ |
| Care outside the home | 1. a. Was the child taken for outside care?   b. If no, why not? | Yes…………………………………………………………………  No…………………………………………………………………  Don’t Know……………………………………………………  ………………………………………………………………………….………………………………………………………………………….…………………………………………………………… | □  □  □ |
|  | 1. How much time after the first symptom was recognised was the child taken outside of the home for care? | Days………………………………………………………………  Don’t know…………………………………………………… | □□  □ |
| (cross stage) | 1. a. Did the child receive any treatment before he/she died?   b. If no, why did the child not receive any treatment? (Select all that apply) | Yes…………………………………………………………………  No…………………………………………………………………  Don’t Know……………………………………………………  Did not seem sick enough………………………………  Thought illness would get better on its own…  Sudden death, no time for treatment……………  Did not know what to do………………………………  No time, too busy…………………………………………  Not enough money for consultation……………..  Transport too expensive………………………………  Too far to travel……………………………………………  Needed permission of another person e.g. husband/father/mother etc, and they were not available/refused…………………………………..  Nobody to go with the child…………………………  Nobody to care for other children at home….  No drugs at health care facility…………………….  Staff attitudes poor………………………………………  Not enough privacy………………………………………  Too little time with health worker………………..  Queue too long/wait too long………………………  Western care could do nothing for illness…….  Other (specify)………………………………………………  Don’t know…………………………………………………… | □  □  □  □  □  □  □  □  □  □  □  □  □  □  □  □  □  □  □  □  □  □ |

| Care outside the home | 1. Specify care sought in chronological order | | | | | | |
| --- | --- | --- | --- | --- | --- | --- | --- |
|  | Type of Provider  1.___________  2.___________  3.___________  4.___________ | When?  Day__________  Day__________  Day__________  Day__________ | | Facility name:  _____________  _____________  _____________  _____________ | Village  __________  __________  __________  __________ | District:  _________  _________  _________  _________ | |
| Receives (quality) care from the healthcare provider | 1. How long after you arrived at the care provider was treatment given? | | Immediately…………………………………………………  Hours……………………………………………………………  Don’t know…………………………………………………… | | | | □  □□  □ |
| Referral | 1. a. Was the child referred to another place for care?   b. Where were they referred to?  c. What was the reason for referral?  d. Did they reach the place they were referred to?  e. If not why? | | Yes…………………………………………………………………  No………………………………………………....................  Don’t Know…………………………………...................  Hospital…………………………………………………………  Health Centre………………………………………………..  Clinic……………………………………………………………..  Chemist/Pharmacy………………………………………..  Traditional Healer…………………………………………  Other (specify)………………………………………………  Don’t know……………………………………………………  Lack of equipment…………………………………………  For better care………………………………………………  Lack of blood…………………………………………………  Lack of drugs…………………………………………………  Lack of oxygen………………………………………………  Other (specify)………………………………………………  Don’t know……………………………………………………  Yes…………………………………………………………………  No………………………………………………....................  Don’t Know……………………………………………………  Died before arrival…………………………………………  Thought it wasn’t necessary………………………….  Hoped/waited for an improvement………………  Lack of money……………………………………………….  Lack of transport…………………………………………..  Other (specify)………………………………………………  Don’t know…………………………………………………… | | | | □  □  □  □  □  □  □  □  □  □  □  □  □  □  □  □  □  □  □  □  □  □  □  □  □  □  □ |
